# Identification of sucrose synthase from *Micractinium conductrix* to favor biocatalytic glycosylation

**DOI:** 10.3389/fmicb.2023.1220208

**Published:** 2023-08-15

**Authors:** Kai Chen, Lei Lin, Ruiqi Ma, Jiajie Ding, Huayi Pan, Yehui Tao, Yan Li, Honghua Jia

**Affiliations:** College of Biotechnology and Pharmaceutical Engineering, Nanjing Tech University, Nanjing, China

**Keywords:** biotransformation, glycosylation, glycosyltransferase, *Micractinium conductrix*, rebaudioside M, sucrose synthase

## Abstract

Sucrose synthase (SuSy, EC 2.4.1.13) is a unique glycosyltransferase (GT) for developing cost-effective glycosylation processes. Up to now, some SuSys derived from plants and bacteria have been used to recycle uridine 5′-diphosphate glucose in the reactions catalyzed by Leloir GTs. In this study, after sequence mining and experimental verification, a SuSy from *Micractinium conductrix* (*Mc*SuSy), a single-cell green alga, was overexpressed in *Escherichia coli*, and its enzymatic properties were characterized. In the direction of sucrose cleavage, the specific activity of the recombinant *Mc*SuSy is 9.39 U/mg at 37°C and pH 7.0, and the optimum temperature and pH were 60°C and pH 7.0, respectively. Its nucleotide preference for uridine 5′-diphosphate (UDP) was similar to plant SuSys, and the enzyme activity remained relatively high when the DMSO concentration below 25%. The mutation of the predicted *N*-terminal phosphorylation site (S31D) significantly stimulated the activity of *Mc*SuSy. When the mutant S31D of *Mc*SuSy was applied by coupling the engineered *Stevia* glycosyltransferase UGT76G1 in a one-pot two-enzyme reaction at 10% DMSO, 50 g/L rebaudioside E was transformed into 51.06 g/L rebaudioside M in 57 h by means of batch feeding, with a yield of 76.48%. This work may reveal the lower eukaryotes as a promising resource for SuSys of industrial interest.

## 1. Introduction

Glycosylation of secondary metabolites with diverse structures, such as flavonoids and terpenoids, has a profound impact on their solubility, stability, or bioactivity (Xiao et al., 2014; Schwab et al., 2015). The majority of glycosylation reactions in nature are catalyzed by glycosyltransferases (GTs), and the representative Leloir GTs with exceptional regio- and stereoselectivity, as well as a broader substrate diversity, are considered the most promising biocatalysts for industrial applications (De Bruyn et al., 2015). A crucial prerequisite is to solve the limited availability of nucleotide-activated sugar donors.

Sucrose synthase (SuSy, EC 2.4.1.13) belongs to the glycosyltransferase-4 subfamily (GT-4), which can catalyze the reversible reaction of sucrose synthesis and cleavage (Stein and Granot, 2019). A large number of studies have shown that the activity of SuSy depended on pH value (Bungaruang et al., 2013; Gutmann and Nidetzky, 2016). At pH 7.5–9.5, it displays optimal activity in the direction of sucrose synthesis, while acidic pH promotes the reverse reaction and decomposes sucrose at pH 5.5–7.5 to produce nucleoside diphosphate (NDP) glucose and fructose. Recently, using sucrose to recover the “donor” uridine 5′-diphosphate (UDP) glucose (UDP-Glc) by combining SuSy with Leloir GT (SuSy-GT) has aroused considerable interest in the development of biocatalytic glycosylation process, because the glycosylation of most known conjugates by Leloir GT requires the participation of UDP-Glc (Gutmann et al., 2017; Schmölzer et al., 2017; Nidetzky et al., 2018). In the SuSy-GT cascade reaction, a UDP cycle is created using sucrose and SuSy which makes UDP-Glc continuously regenerated as an expedient donor for glucoside production. More than that, the removal of UDP, a product inhibitor of Leloir GT, will enhance the glycosylation efficiency (Terasaka et al., 2012).

It is known that SuSy has a broad substrate spectrum for different NDP “acceptors” (Kulmer et al., 2017). In the past five decades, more attention has been focused on plant SuSys with UDP preference, which is conducive to the production of UDP-Glc (Schmölzer et al., 2016; Zhang et al., 2019). Prokaryotic SuSys are diversified in nucleotide substrate preference, such as some characterized SuSys from *Thermosynechococcus elongatus* (*Te*SuSy), *Nitrosomonas Europaea* (*Ne*SuSy), *Acidithiobacillus caldus* (*Ac*SuSy), and *Denitrovibrio acetiphilus* (*Da*SuSy), which are more inclined to use adenosine 5′-diphosphate (ADP) as nucleotide (Figueroa et al., 2013; Diricks et al., 2015). But bacterial SuSys showed better thermostability than plant SuSys, which could be more suitable for application in large-scale industrial production by increasing reaction temperature to avoid microbial contamination (Desmet et al., 2012; Diricks et al., 2016). To obtain a bacterial SuSy variant suitable for UDP-Glc regeneration during glycosylation reactions, the affinity of *Ac*SuSy for UDP has been significantly improved by introducing plant residues at positions of a putative nucleotide binding motif (QN motif) (Diricks et al., 2016). Comparison was made between the L637M-T640V double mutant of *Ac*SuSy and the SuSy from Glycine max (*Gm*SuSy). As a result, fitness in terms of kinetics, expressed by the relatively low *K*_*m*_ values for UDP and sucrose, superseded enhanced thermostability in bacterial SuSys as the selection criterion, which made plant SuSys the strongly preferred choice (Gutmann et al., 2017).

However, most of the receptors to be glycosylated have poor water solubility, so dimethyl sulfoxide (DMSO) is added as cosolvent to increase the solubility of the substrate (Pei et al., 2019; Chu et al., 2021; Tao et al., 2023). The low stability and intolerance to organic cosolvents of plant SuSys have limited the further application of such SuSy-GT cascades (Schmölzer et al., 2016). Therefore, mining or engineering of SuSys for high robustness has become one of the important tasks for biocatalytic glycosylation. Undoubtedly, with the rapid development of sequencing technology and data mining algorithms, more SuSys would be uncovered from the ever-increasing numbers of sequences with improved properties (Zhang et al., 2019; Zhao et al., 2023). Herein, by sequence mining, we focused on SuSys from lower eukaryotes like green algae, and their characteristics are still little known. A candidate SuSy-encoding sequence derived from *Micractinium conductrix* (*Mc*SuSy) was overexpressed in *Escherichia coli* BL21 (DE3), and the recombinant SuSy was characterized. The site-directed mutagenesis was conducted at the predicted *N*-terminal phosphorylation site (S31) of *Mc*SuSy. Furthermore, the S31D mutant with enhanced activity and the engineered glycosyltransferase UGT76G1 (UGT76G1_S195Q) from *Stevia rebaudiana* were co-expressed in *E. coli* to construct a SuSy-GT reaction. In the biotransformation of rebaudioside E (RebE, PubChem CID 72710721) into rebaudioside M (RebM, PubChem CID 92023628) (Yu et al., 2022), the issue of low solubility of the intermediate product rebaudioside D (RebD, PubChem CID 71773169) was alleviated by addition of DMSO, to obtain an enhanced product yield.

## 2. Materials and methods

### 2.1. Sequence mining

Two SuSy sequences from *Anabaena* sp. PCC 7119 (*An*SuSy, CAA09297) and *Melioribacter roseus* (*Mr*SuSy, AFN74551) were used as templates for BLAST search in NCBI.^1^ The resulting 20,000 sequences were downloaded for further analysis (in April 2020). Multiple sequence alignment was performed using MAFFT-7.037 (Katoh et al., 2002) or ClustalW^2^ with default parameters. After removing redundancy, three putative SuSys from algae, including *Micractinium conductrix* (*Mc*SuSy, PSC73946) and *Chara braunii* (*Cb*SuSy1, GBG73881; *Cb*SuSy2, GBG70160), were selected from the sequences with conserved residues G302, G303, H438, R580, L581, K585, Q648, N654, E675, and E683 (Zheng et al., 2011; Diricks et al., 2015; Wu et al., 2015), and used as candidates in the subsequent experiments. The residue number refers to the sites of SuSy from *Arabidopsis thaliana* (*At*SuSy1, CAA50317) in the multiple sequence alignment.

Solubility predictions from sequences were performed using Protein-sol^3^ (Hebditch et al., 2017). The translated protein sequences of *Mc*SuSy, *Cb*SuSy1, and *Cb*SuSy2 were used to construct a phylogenetic tree using MEGA 7.0 (Kumar et al., 2016) with the known SuSys from *G. max* (*Gm*SuSy, AAC39323), *A. thaliana* (*At*SuSy1,CAA50317; *At*SuSy3,CAB80721), *S. tuberosum* (*St*SuSy1, AAA33841), *D. acetiphilus* (*Da*SuSy, ADD69694), *A. caldus* (*Ac*SuSy, AIA55343), *N. europaea* (*Ne*SuSy, CAD85125), *M. roseus*, *T. elongatus* (*Te*SuSy, BAC08600), and *Anabaena* sp. PCC 7119 by using the neighbor-joining method (Saitou and Nei, 1987). Motifs were found by MEME (Bailey and Elkan, 1994) according to the result of sequence alignment and displayed by WebLogo^4^ (Crooks et al., 2004).

To predict the phosphorylation sites of SuSys, protein sequences of *Mc*SuSy, *Gm*SuSy, and the SuSy from *Zea mays* (*Zm*SuSy) were submitted to NetPhos 3.1 Server^5^ (Blom et al., 1999).

### 2.2. Structure modeling and molecular docking

The homology model of *Mc*SuSy was constructed using the YASARA program (Krieger et al., 2002). The structures of UDP and sucrose were obtained from ZINC database.^6^ To construct the complex structure for evaluating the interaction between the protein and substrates, we tested molecular docking software such as LeDock and AutoDock Vina (Morris et al., 2009; Trott and Olson, 2010; Zhao and Caflisch, 2013) to dock the structure of *At*SuSy1 (PDB ID: 3S27, chain A) with its substrates. The docking results obtained by LeDock have a relatively good reproducibility to the crystal structure of *At*SuSy1, therefore, LeDock was further used to obtain the complex of *Mc*SuSy. UDP was first docked into the active site of *Mc*SuSy, resulting in the structure of *Mc*SuSy with UDP, which was then docked with sucrose. PyMOL (Version 2.4.1, Schrodinger LLC) was used to visualize and analyze the model structures generated, as well as to build illustrative figures.

### 2.3. Plasmid and strain construction

After codon optimization for heterologous expression in *E. coli*, the coding region derived from the putative SuSy mentioned above (Supplementary Data) was synthesized and cloned into pRSFDuet-1 (Novagen) between the restriction endonuclease sites *Nco*I and *Eco*RI by GenScript (Nanjing, China). A 6-histidine tag was added at the *C*-terminus of SuSy. The generated plasmids were named as pRSF-*Mc*SuSy, pRSF-*Cb*SuSy1, and pRSF-*Cb*SuSy2, respectively.

The plasmid pRSF-*Mc*SuSy was used as the template for site-directed mutagenesis by a Mut Express^®^ II Fast Mutagenesis Kit V2 (Vazyme Biotech Co., Ltd., Nanjing, China). The primers used in PCR to produce the plasmid mutants are listed in Supplementary Table 1.

The code-optimized genes coding for UGT76G1_S195Q and the S31D mutant of *Mc*SuSy were synthesized and cloned into the restriction endonuclease sites *Nde*I/*Xho*I and *Nco*I/*Eco*RI of the pRSFDuet-1, respectively (Yu et al., 2022). The obtained plasmid was named pRSF-S31D-S195Q. Then, the coding region of the *Mc*SuSy mutant was replaced by that of *At*SuSy1 in pRSF-S31D-S195Q, giving another plasmid named pRSF-*At*SuSy1-S195Q.

The aforementioned plasmids were, respectively transformed into *E. coli* BL21 (DE3) competent cells (TransGen Biotech, Beijing, China), resulting in the corresponding recombinant strain.

### 2.4. Expression and purification of SuSys

The recombined *E. coli* was first incubated in 5-mL Luria-Bertani medium containing 10 g/L tryptone, 10 g/L NaCl, 5 g/L yeast extract and 50 μg/mL kanamycin, and incubated overnight at 37°C with continuous shaking at 200 rpm. Then, 2% (v/v) of the overnight culture was incubated in shake flasks with 100-mL LB medium containing 50 μg/mL kanamycin to cultivate for about 2 h at 37°C. Isopropyl-β-D-thiogalactopyranoside in a final concentration of 0.1 mM was added when the culture turbidity (OD_600_) reached 0.5–0.6, and then the cultivation was continued at 16°C for another 24 h. The subsequent steps involving purification were performed at 4°C. Cells harvested by centrifugation at 5,289 *g* for 5 min, were resuspended in an appropriate lysis buffer [500 mM NaCl and 10% glycerine (v/v) in 20 mM sodium phosphate buffer, pH 8.0] and disrupted by sonication. After centrifuge twice at 6,665 *g* for 15 min, the 6 Histidine-tagged proteins in the supernatant (crude extract) were purified by immobilized metal-affinity chromatography (IMAC) using a Ni-charged resin (L00683, GenScript, Nanjing, China). The recombination proteins were eluted from the column by stepwise imidazole gradient. Fractions with SuSy activity were pooled and concentrated in an Amicon^®^ Ultra-15 Centrifugal Filter Unit with an Ultracel-30 membrane (Merck Millipore Ltd., Ireland), and the buffer was exchanged to 50 mM HEPES (N-2-hydroxyethylpiperazine-N-ethane-sulphonicacid)-NaOH (pH 7.0). The protein expression and the purity of recombinant enzymes were analyzed using SDS-PAGE.

### 2.5. Enzyme assays of SuSys

The activities of purified SuSys in the sucrose cleavage direction were measured with the standard reaction mixture containing 50 mM HEPES-NaOH (pH 7.0), 2 mM UDP, 200 mM sucrose, and appropriate amount of purified enzyme in a final volume of 50 μL. Reactions were carried out at 37°C for 5 min and stopped by heating at 95°C for 2 min, and control experiments were performed immediately to check the decomposition of sucrose by the heat treatment. The product fructose was determined by the reduction of NAD^+^ at 340 nm following the addition of a 150-μL solution that contained 50 mM HEPPS-NaOH (pH 7.0), 1 mM MgCl_2_, 1 mM NAD^+^, 1 mM ATP, 1 μg hexokinase, 1 μg P-glucose isomerase, and 1 μg glucose-6-P dehydrogenase (Figueroa et al., 2013). The SuSy activity of crude extract in the sucrose cleavage direction was measured by 3, 5-dinitrosalicylic acid (DNS) method (Miller, 1959). Appropriate amount of crude extract was added to 100 μL of assay solution containing 50 mM HEPES (pH 7.0), 200 mM sucrose and 5 mM UDP, and reactions were carried out at 37°C for 20 min and quenched by heating. Control experiments were performed to check decomposition of sucrose by the heat treatment. One unit of SuSy activity is defined as the amount of enzyme that releases 1 μmol of reducing sugars per minute under the specified conditions.

The pH optimum of SuSy activity in the cleavage direction was determined in the pH ranging from 5.0 to 8.5 at 0.5 pH unit intervals. Buffers used were 50 mM MES-HCl (pH 5.0–7.0) and HEPES-NaOH (pH 7.0–8.5). The initial concentrations of sucrose and UDP in the reaction mixture were 200 and 2 mM, respectively.

The temperature profiles were obtained by determining the SuSy activity in the direction of sucrose cleavage from 20 to 70°C. In the evaluation of thermal stability, the enzyme was pre-incubated in 50 mM HEPES buffer (pH 7.0) for 15 min from 30 to 60°C without any substrates, alternatively, with the addition of 200 mM sucrose. After the incubation, the residual activity in the sucrose cleavage direction was checked with the standard assay described above.

The influence of divalent metal ions on SuSy was investigated by measuring the activity in the presence of 2 mM of MgCl_2_, MnCl_2_, CaCl_2_, NiCl_2_, CuCl_2_, BaCl_2_, ZnCl_2_, or Ethylene diamine tetraacetic acid (EDTA). The influence of Dimethyl sulfoxide (DMSO) on SuSy was investigated by measuring the activity in the presence of different concentrations of DMSO.

The kinetic parameters for sucrose varying from 50 to 600 mM at a constant concentration of 2 mM UDP and for UDP varying from 0.05 to 5 mM at a constant concentration of 200 mM sucrose were measured at 37°C in 50 mM HEPES buffer (pH 7.0). Apparent *K*_*m*_ and *V*_*max*_ values were calculated by non-linear regression of the Michaelis–Menten equation using OriginPro Learning Edition.

All reactions were conducted in triplicate. The relative activity (%) was calculated in terms of that of the maximum activity (100%). Coupled enzymes used for SuSy activity assays were purchased from Shanghai yuanye Bio-Technology Co., Ltd., and all the other reagents were analytical grade and commercially available.

### 2.6. SuSy-GT reactions

To explore the application potential of *Mc*SuSy, we established SuSy-GT reactions to convert RebE into RebM. The reaction mixtures (5 mL) contained 15, 20 or 30 g/L of RebE, 150, 200 or 300 g/L of sucrose, 0–30% (v/v) DMSO, potassium phosphate buffer (50 mM, pH 7.2), and appropriate amount of the crude extract prepared from *E. coli* BL21 (pRSF-S31D-S195Q), or *E. coli* BL21 (pRSF-*At*SuSy1-S195Q), in which expression of two recombinant enzymes was under the same conditions as SuSys, except for the induction for 36 h. For the convenience of description, the reactions were named S31D-S195Q and *At*SuSy1-S195Q, respectively. The reaction was incubated at 40°C and 200 rpm for 24 h, and the samples were taken and heated for 10 min at 95°C. In the fed-batch reactions, the powder of RebE and sucrose, which kept the mass ratio at 1:10, was added at the specified time. The enzyme activity of SuSy was measured by the DNS method as described above, but in potassium phosphate buffer (pH 7.2). The GT activity of UGT76G1_S195Q was measured as previously described using RebE and UDP-Glc as the substrates (Yu et al., 2022). After being properly diluted and filtered, the concentrations of RebD and RebM in the reaction mixtures were determined by HPLC as previously described (Yu et al., 2022).

## 3. Results

### 3.1. Sequence screening

In the sequence mining, the prokaryote-derived SuSy templates *An*SuSy and *Mr*SuSy were used for sequence collection and the sequences without conservative residues G302, G303, H438, R580, L581, K585, and E675 (Zheng et al., 2011), and residues Q648, N654, and E683 that contribute to UDP-Glc binding were removed (the residue number refers to *At*SuSy1 in the multiple sequence alignment) (Diricks et al., 2015; Wu et al., 2015). As a result, only 14 sequences from lower eukaryote sources like green algae remained together with a large number of the putative plant SuSys. These 14 sequences fall into *Helicosporidium* sp. (KDD76488), *M. conductrix* (PSC73946), and *C. braunii* (GBG70160, GBG73779, GBG73781, GBG73784, GBG73881, GBG76990, GBG86653, GBG89666, GBG89682, GBG92531, GBG92534, and GBG92536). The sequence from *Helicosporidium* sp. was ruled out for heterologous expression due to the low predicted protein solubility (0.389). Two sequences (GBG73881, GBG70160) with 69.48% identity (called *Cb*SuSy1 and *Cb*SuSy2, respectively) from those putative SuSys from *C. braunii*, along with the one from *M. conductrix* (*Mc*SuSy) was selected and synthesized for expression in *E. coli*. Enzyme activities of the crude extracts containing *Mc*SuSy and *Cb*SuSy2 were around 15.5 and 5.5 mU/mg total protein, respectively. However, it was difficult to detect the SuSy activity of *Cb*SuSy1. Therefore, *Mc*SuSy was chosen for further study of enzymatic properties.

### 3.2. Purification and enzymatic properties of *Mc*SuSy

*Mc*SuSy fused with a *C*-terminal histidine-tag that was overexpressed in *E. coli* BL21 (pRSF-*Mc*SuSy), was purified by Ni-NTA affinity chromatography (Supplementary Table 2). In SDS-PAGE, it corresponded to a band close to 97.2 kDa (Supplementary Figure 1). The specific activity of recombinant *Mc*SuSy is 9.39 U/mg at 37°C and pH 7.0. The kinetic constants for the cleavage reaction were determined: *K*_*m*_ (UDP) 0.14 mM; *K*_*m*_ (sucrose) 90 mM (Supplementary Figure 2).

**FIGURE 1:**
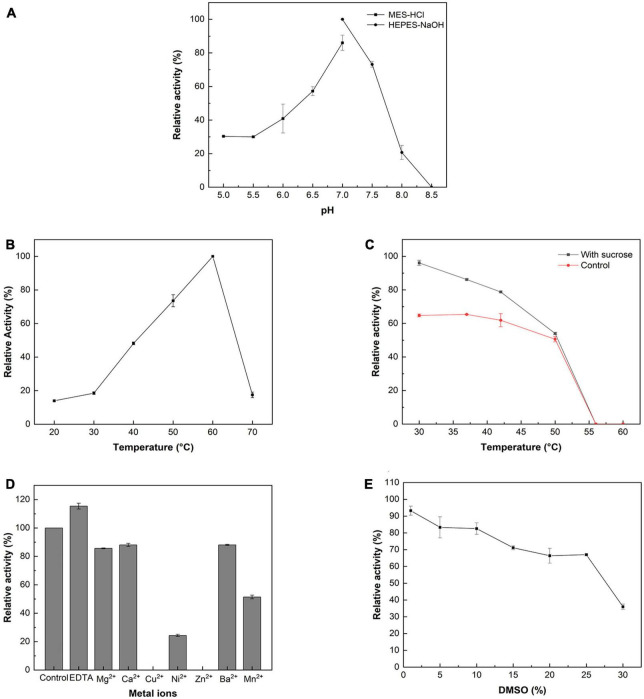
Enzymatic properties of the recombinant *Mc*SuSy. **(A)** pH profile; **(B)** Temperature profile; **(C)** Thermal stability; **(D)** Effect of metal ions on the activity of *Mc*SuSy; **(E)** Effect of DMSO on the activity of *Mc*SuSy. The data are presented as the means ± standard deviation of triplicates. The relative activity (%) was calculated in terms of that of the maximum activity (100%).

According to the pH profile (Figure 1A), *Mc*SuSy reached its maximum activity at pH 7.0 and showed high enzymatic activity (>70% of maximum value) between pH 7 and pH 7.5. From pH 6.0–7.5, its activity was still higher than 40% of the maximum value, while the activity was undetectable at pH 8.5. The optimal temperature of *Mc*SuSy was 60°C ranging from 20 to 70°C (Figure 1B). After incubating the enzymes for 15 min between 30 and 60°C with or without sucrose, the thermostability of *Mc*SuSy was determined by measuring the residual activity. The enzyme remained stable up to 42°C after 15 min of incubation without substrates, but its activity sharply decayed beyond 50°C (Figure 1C). It is worth mentioning that sucrose is known to act as a stabilizing agent, and the result found that sucrose plays a positive role in maintaining enzyme activity. Addition of 200 mM sucrose enhanced enzyme activity by about 2 U/mg at the lower incubation temperature (30, 37°C) compared to the case without sucrose.

In terms of the effect of divalent metal ions, adding 2 mM of Mg^2+^, Ca^2+^, Cu^2+^, Ni^2+^, Zn^2+^, Ba^2+^, and Mn^2+^, the activities of *Mc*SuSy were observed to decrease (Figure 1D). It was especially strongly inhibited by Cu^2+^ and Zn^2+^, resulting in undetectable activity, since these ions may influence the interaction with clusters of histidine on the protein surface (Elling, 1995). In the presence of ethylene diamine tetraacetic acid (EDTA), a chelating agent, the activity of *Mc*SuSy was promoted by removing trace metal ions.

The effect of DMSO on *Mc*SuSy activity was investigated by adding different concentrations of DMSO (v/v) in the assay mixtures (Figure 1E). When the DMSO concentration increased from 1 to 25%, the enzyme activity decreased gradually. At 10% DMSO, *Mc*SuSy maintained a high activity (>80% of maximum value), and while adding 30% DMSO reduced enzyme activity to 36%.

### 3.3. UDP preference of *Mc*SuSy

**FIGURE 2:**
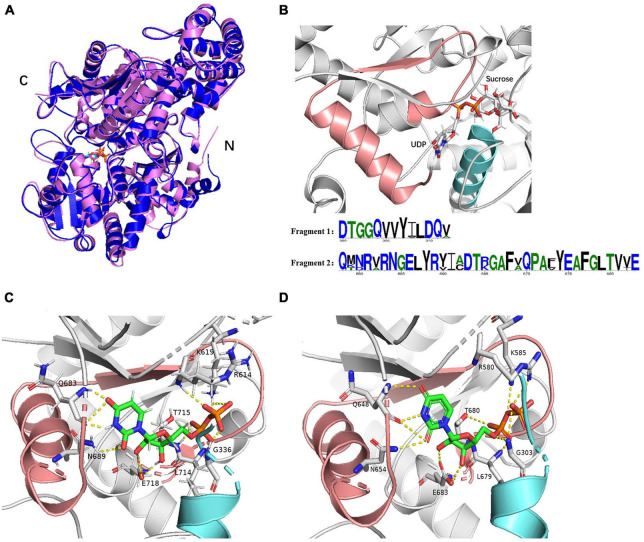
The structure models of *Mc*SuSy and *At*SuSy1. **(A)** Ribbon drawing of the structure of *Mc*SuSy (blue) aligned with that of *At*SuSy1 (purple). **(B)** The *Mc*SuSy complex with two conserved sequence fragments: residues 300–312 (light blue) and residues 648–683 (pink). The residue number refers to the sites of *At*SuSy1 in the multiple sequence alignment. **(C,D)** The UDP binding sites of *Mc*SuSy and *At*SuSy1, respectively.

SuSys, like *St*SuSy (*S. tuberosum* L) and SuSy*Ne* (*N. europaea*) show high flexibility for nucleoside diphosphates in the cleavage reaction (Römer et al., 2004; Diricks et al., 2015). Plant SuSys preferentially utilize UDP as an acceptor nucleotide, while bacterial SuSys prefer ADP (Table 1). The *K*_*m*_ value of *Mc*SuSy for UDP is 0.14 mM, indicating that *Mc*SuSy has a higher affinity for UDP. And it was difficult to determine the enzyme activity under the same conditions when ADP was the glycosyl receptor. Homology modeling was carried out using the crystal structure of *At*SuSy1 (PDB ID: 3S27) as a template, which has 55.17% sequence identity with *Mc*SuSy, and the complex was obtained by substrate docking using LeDock. The observed secondary structure of *Mc*SuSy is very similar to that of the *At*SuSy1 monomer (Figure 2A). Two sequence fragments, residues 333 to 345 and residues 683 to 718, were found in the active site of *Mc*SuSy, which are highly conserved in plant SuSys (Figure 2B). To be specific, the residues 333 to 345 of *Mc*SuSy (light blue), corresponding to the residues 300 to 312 of *At*SuSy1, participate in the binding of fructose and G336 (G303 in *At*SuSy1) also interacts with β-phosphate of UDP by forming hydrogen bonds (Figures 2C, D; Zheng et al., 2011; Wu et al., 2015). The residues 683 to 718 of *Mc*SuSy (pink) corresponding to the residues 648 to 683 of *At*SuSy1 belong to the nucleotide-binding domain, which contains the “QN” motif, playing a significant role in the nucleotide preference of SuSy (Wu et al., 2015; Diricks et al., 2016). In particular, the two amino acids Q683 and N689 (Q648 and N654 in *At*SuSy1) are highly conserved in plant SuSys, while in bacteria the residues are highly variable (Diricks et al., 2016). For example, R636 and A642 in the *N. europaea* create a more spacious binding site for the preference toward the bulkier ADP substrate (Wu et al., 2015). As shown in Figures 2C, D, the UDP moieties bind of *Mc*SuSy are the same way as *At*SuSy1, especially in the indicated “QN” motif, which also implies a similar preference for nucleotide bases (Zheng et al., 2011).

**TABLE 1:** Kinetic parameters of SuSys for UDP in the sucrose cleavage direction.

SuSys*^a^*	Conditions	*K*_*m*_ (mM)	*V*_*max*_ (U/mg)	*K*_*cat*_ (s^–1^)	*K*_*cat*_*/K*_*m*_ (mM^–1^s^–1^)	References
*At*SuSy1	37°C, pH 7.0	0.39	585	907	2325.6	Baroja-Fernández et al., 2012
*At*SuSy3	37°C, pH 7.0	0.25	950	1,470	5,880	Baroja-Fernández et al., 2012
*Gm*SuSy	30°C, pH 7.5	0.13	n.a.	9.3	71.5	Bungaruang et al., 2013
*St*SuSy1	30°C, pH 7.5	0.2	0.13	0.2	1	Sauerzapfe et al., 2008
*Mc*SuSy	37°C, pH 7.0	0.14	10.4	17.3	123.6	This study
*Mc*SuSy_S31D	37°C, pH 7.0	0.092	11.4	19	206.5	This study
*AcSuSy*	60°C, pH 7.0	7.8	96.7	82	10.5	Diricks et al., 2015
*Ne*SuSy	60°C, pH 7.0	0.69	67.7	103.8	150.4	Diricks et al., 2015
*Te*SuSy	37°C, pH 7.0	1.3	2.2	3.5	2.7	Figueroa et al., 2013
*An*SuSy	30°C, pH 6.5	0.8	2.8	n.a.	n.a.	Curatti et al., 2000

^a^ The SuSys were overexpressed in *E. coli*; n.a., not available.

### 3.4. Mutation of the predicted *N*-terminal phosphorylation sites of *Mc*SuSy

**FIGURE 3:**
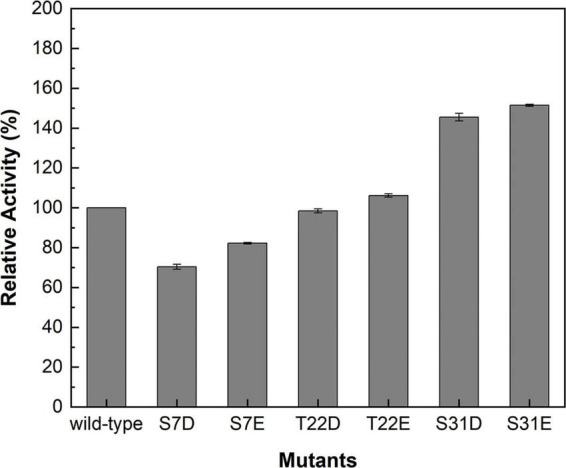
Relative activities of the crude extracts containing the wild-type and mutants of *Mc*SuSy.

Studies have demonstrated that phosphorylation affected the catalytic activities of SuSys in sucrose cleavage, which may adjust the apparent affinity of the enzyme for sucrose and UDP to activate the formation of UDP-Glc and fructose from sucrose plus UDP (Huber et al., 1996; Takeda et al., 2017). Three residues including S7, T22, and S31 at the *N*-terminus of *Mc*SuSy (Supplementary Table 3), which were predicted reliably as phosphorylation sites by NetPhos 3.1 Server, were mutated into two different acidic amino acid residues Asp (D) or Glu (E), respectively. As shown in Figure 3, the enzyme activity of crude extracts from the S7D, S7E, and T22D mutants declined slightly, and those from both S31D and S31E mutants increased significantly (more than 40% compared with wild-type *Mc*SuSy). After purification, the kinetic parameter of the S31D mutant (*Mc*SuSy_S31D) was determined (Supplementary Figure 3). The *K*_*m*_ values of *Mc*SuSy_S31D were 0.092 mM and 88 mM for UDP and sucrose, respectively. A *K*_*m*_ drop of more than 34.3% indicated an increased affinity for UDP compared with the wild type of *Mc*SuSy (Table 1).

### 3.5. Production of RebM from RebE by the SuSy-UGT reaction

**FIGURE 4:**
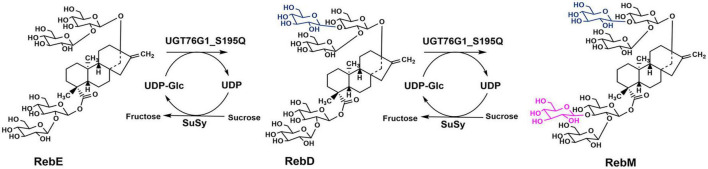
Schematic diagram of RebE glycosylation reaction catalyzed by UGT76G1_S195Q and SuSy.

**FIGURE 5:**
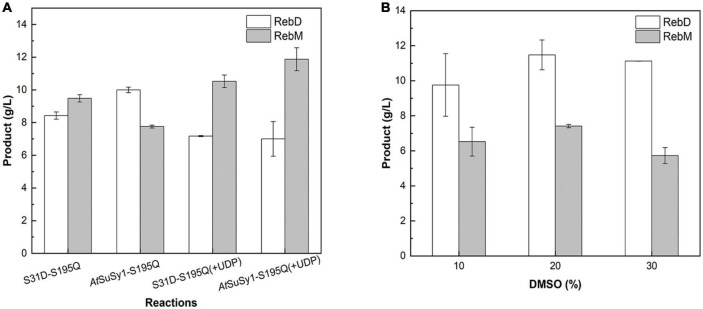
Production of RebM from RebE in the SuSy-UGT reactions. **(A)** The reactions catalyzed by *At*SuSy1-S195Q and S31D-S195Q at 40°C for 24 h, with the crude extract (1.21 U/mL UGT in ∼6 mg/mL of total protein), 20 g/L RebE, and 200 g/L sucrose, with or without 1 mM UDP. **(B)** The reactions catalyzed by S31D-S195Q at 40°C for 8 h, with the crude extract (6 mg/mL of total protein), 20 g/L RebE, 200 g/L sucrose, 1 mM UDP, and different concentrations of DMSO. Data are plotted as means ± standard deviation of duplicates.

The engineered glucosyltransferase UGT76G1_S195Q derived from *S. rebaudiana*, was recently reported that can efficiently convert RebE into RebM via a two-step continuous glycosylation (Figure 4; Yu et al., 2022). In the present study, *Mc*SuSy_S31D and UGT76G1_S195Q prepared from *E. coli* BL21 (pRSF-S31D-S195Q) were coupled to form a SuSy-GT system (S31D-S195Q). A control experiment (*At*SuSy1-S195Q) was performed under the same conditions using UGT76G1_S195Q and *At*SuSy1, which was widely applied in various glycosylation reactions (Chen T. Y. et al., 2021; Chu et al., 2021; Ping et al., 2022). The cascade reactions were carried out at pH 7.2 and 40°C, and the mass ratio of RebE to sucrose was set to 1:10 (Yu et al., 2022). The GT activity in *At*SuSy1-S195Q was apparently higher than that in S31D-S195Q (Supplementary Table 5), suggesting the expression difference of UGT76G1_S195Q existed when SuSys were different, although the genes encoding GT and SuSy were cloned into the same sites of plasmid. The SuSy activity in *At*SuSy1-S195Q was 1.85 folds of that in S31D-S195Q (Supplementary Table 5). The reaction mixture changed from clear to milky white after reaction of 3 h, and the intermediate product RebD started to accumulate as visible white precipitate. After 24 h of reaction (Figure 5A), the products RebD and RebM synthesized by S31D-S195Q were 8.43 and 9.48 g/L, respectively, where the RebM yield was 35.5%. The concentration of RebM in *At*SuSy1-S195Q was 7.75 g/L, which was 18.2% lower than that in S31D-S195Q. Considering that UDP-Glc and UDP from the cell lysates are in limited amounts and prone to degradation for a long time of reaction (Baroja-Fernández et al., 2012; Gutmann and Nidetzky, 2016), an extra 1 mM UDP was added to the initial solutions, which promoted RebM production in both reactions. The concentrations of RebM in S31D-S195Q and *At*SuSy1-S195Q were 10.52 and 11.87 g/L, respectively. The RebD concentrations in both reactions kept almost the same (around 7 g/L).

**FIGURE 6:**
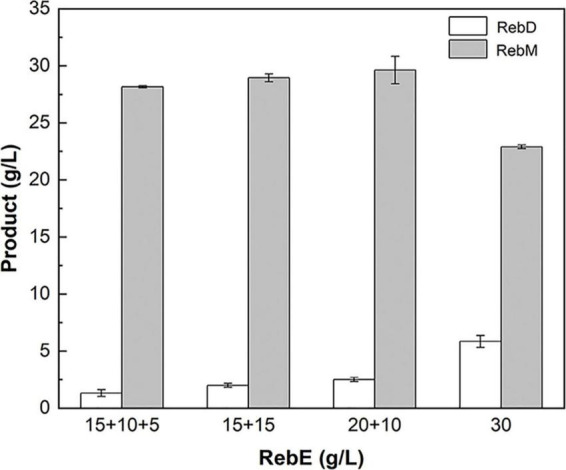
Fed-batch synthesis of RebM catalyzed by S31D-S195Q. The reactions were carried out at 40°C for 24 h, with the crude extract (2.14 U/mL UGT and 3.93 U/mL SuSy in 10 mg/mL of total protein), RebE, sucrose (10 times of RebE), 10% DMSO, and 1 mM UDP. A: the initial concentration of RebE was 15 g/L, and the RebE powder equivalent to 10 and 5 g/L, was added at 6 and 10 h, respectively; B:the initial concentration of RebE was 15 g/L, and the RebE powder equivalent to 15 g/L was added at 10 h; C: the initial concentration of RebE was 20 g/L, and the RebE powder equivalent to 10 g/L was added at 10 h; D: the initial concentration of RebE was 30 g/L. When feeding, sucrose was added along with RebE to keep the constant radio of 10:1. Data are plotted as means ± standard deviation of duplicates.

The poor water solubility of RebD (Zhang et al., 2021) may lead to low RebD concentration in the aqueous solutions, which would limit the rate of the second glycosylation reaction that RebD was converted into RebM by UGT76G1_S195Q. Since addition of DMSO would improve the RebD solubility, the influence of DMSO concentrations on the activities of *Mc*SuSy_S31D and *At*SuSy1 of the crude extracts were investigated (Supplementary Figure 4). In the range from 1 to 30% DMSO, *Mc*SuSy_S31D remained higher residual activity than that of *At*SuSy1. Therefore, 10, 20, and 30% (v/v) DMSO was added to the S31D-S195Q reactions (Figure 5B). After reaction of 8 h, the total yield of RebD and RebM was the highest in the reaction mixtures containing 20% DMSO, and the RebD concentration kept more than 10 g/L in all the systems. In view that *Mc*SuSy maintained more that 80% of maximum activity at 10% DMSO (Figure 1E and Supplementary Figure 4), the subsequent RebM synthesis from 30 g/L RebE was performed at 10% DMSO (Figure 6). Compared with RebM (22.90 g/L) in the 24-h batch production (D), the fed-batch reactions (A, B, and C) obtained more RebM (28.17, 28.95, and 29.62 g/L, respectively), reaching the yields of above 70%. Subsequently, 20 g/L RebE and 200 g/L sucrose were used as the initial substrates, and equivalent to 10 g/L RebE and 10-fold sucrose were fed at 10, 24, and 37 h. Incubation at 40°C continued for another 20 h. The resultant product of interest in the 57-h reaction mixture was 51.06 g/L, with a RebM yield of 76.48%.

## 4. Discussion

In the present study, the prokaryotic *An*SuSy and *Mr*SuSy were used as templates for sequence collection, and the homology, as well as the active site of the reported SuSys, were also considered in the sequence screening process. Particularly, the sequences that have the conserved residues contributing to UDP-Glc binding, corresponding to Q648, N654, and E683 in *At*SuSy1 remained (Diricks et al., 2015; Wu et al., 2015). The phylogenetic tree shows the classification and evolutionary relationship of three selected sequences (*Mc*SuSy, *Cb*SuSy1, and *Cb*SuSy2) from the algae and several other characterized SuSys from plants and bacteria (Supplementary Figure 5). They are close to those from plants, falling in the Eukaryotic group, and share the common conserved active site residues in retaining GT-B glycosyltransferases (Supplementary Table 4), which was known from the multiple sequence alignment. What we focused on was *Mc*SuSy, which was heterologously expressed in *E. coli* with higher activity than *Cb*SuSy1 and *Cb*SuSy2. Lower pH values are known to promote the cleavage reaction of SuSys, yielding NDP-glucose and fructose, and with the increasing of the pH, NDP-glucose synthesis is disfavored (Bungaruang et al., 2013; Gutmann and Nidetzky, 2016; Schmölzer et al., 2016). While the *Mc*SuSy displayed the highest activity at pH 7.0 in sucrose degradation (Figure 1A), which is different from other sources of SuSys preferring to hydrolyze sucrose at acidic pH. And it is suitable to apply in SuSy-GT cascade reactions where Leloir GT having the optimal neutral or slightly basic pH.

**FIGURE 7:**
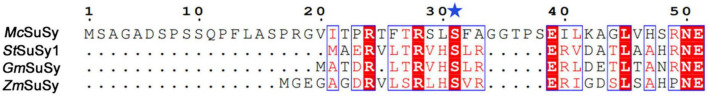
Sequence alignment of the selected SuSys at *N*-terminus. The site corresponding to S31 in *Mc*SuSy, S11 in *St*SuSy1 and *Gm*SuSy, and S15 in *Zm*SuSy is marked with a blue star.

In addition, the plant SuSy is a known phosphoserine-containing enzyme (Komina et al., 2002). One distinctive characteristic feature of SuSys is that phosphorylation of the *N*-terminus at the major phosphorylation site in plants contributes to the fine-tuning of enzyme activity and may be responsible for changes in membrane binding (Komina et al., 2002; Hardin et al., 2004). In contrast, interestingly, the *N*-terminal sequence alignment of prokaryotic SuSys shows that a highly conserved motif was found in cyanobacteria SuSys as a putative phosphoacceptor, but for non-cyanobacteria SuSys, there is no definite motif to distinguish (Schmölzer et al., 2016). Previous studies have shown that phosphorylation or introducing the negative charge at the *N*-terminal phosphorylation site of plant SuSys, such as at S15 of *Zm*SuSy and S11 of *Gm*SuSy and *St*SuSy1, has affected their catalytic activities in sucrose cleavage (Komina et al., 2002; Hardin et al., 2004; Sauerzapfe et al., 2008). In the *N*-terminal sequence alignment of four SuSys involving *Mc*SuSy, *Gm*SuSy, *StS*uSy1, and *Zm*SuSy, the reported phosphorylation site is conserved (Figure 7) in *Mc*SuSy (S31), which is identical to the predicted results obtained from NetPhos 3.1 Server (Supplementary Table 3). S31D mutation of *Mc*SuSy showed a nearly 1.2-fold increase in the enzyme activity, which suggest that introduction of the negative charge at S31, like phosphorylation, may affect the *N*-terminal conformation and the interactions between adjacent region, thus stimulating the catalytic activity of *Mc*SuSy (Hardin et al., 2004; Zheng et al., 2011). Low *K*_*m*_ values for UDP are beneficial for *in vitro* recycling of UDP-Glc in SuSy-GT coupled systems due to favored sucrose cleavage, and the product can be synthesized with endogenesis UDP. The *K*_*m*_ of *Mc*SuSy for UDP (0.14 mM) is comparable to that of most plant SuSys (Table 1). Its S31D mutant reached an even lower *K*_*m*_ at 0.092 mM, the lowest in those of SuSys expressed in *E. coli*, as listed in Table 1. But both *K*_*cat*_ values for UDP were much the same (17.3 s^–1^ vs. 19 s^–1^). Although the catalytic efficiency (*K*_*cat*_/*K*_*m*_) of the S31D mutant was increased by over 67% compared with the wild type, it was still inferior to that of the two *Arabidopsis*-derived SuSys (Table 1). The affinity for sucrose, indicated by the *K*_*m*_ values (∼90 mM), was much worse than that of plants, which implies the demand for high-concentration sucrose in the reactions and that *Mc*SuSy would not be inhibited by high concentrations of sucrose as well. However, the catalytic efficiency of *Mc*SuSy_S31D was higher than all of the reported prokaryotic SuSys (Table 1).

RebM, originally identified from *S. rebaudiana*, is a next-generation of non-calorie sweetener in the approved list of food additivities (Sun et al., 2021). With the growing demands for sustainability, people have aroused great interest in obtaining safe and “natural” products through microbial production. The SuSy-GT cascade reactions have previously been used in enzymatic glycosylation of steviol glycosides to produce RebA, RebE, and RebD (Wang et al., 2016; Chen et al., 2018, 2020; Chen L. L. et al., 2021), and now to produce RebM (Guo et al., 2022; Yu et al., 2022). The strategies of co-immobilizing or fusing the glycosyltransferases have also been applied (Wang et al., 2021, 2023). Guo et al. (2022) used a variant UGT76G1-T284S/M88L/L200A coupled with sucrose synthase *At*SuSy1 in a cascade reaction that produced 23.37 g/L RebM from RebD with a yield of 90.5%. In this study, RebM was synthesis from RebE via a two-step continuous glycosylation as previously reported (Yu et al., 2022). With the aid of SuSy, the sugar donor UDPG for GT can be regenerated from sucrose and the trace UDP (usually 1–2 mM) (Mao et al., 2006) in the crude extract that was prepared from the recombinant cells. In case that the UDP/UDP-Glc in the cell lysate was used for UDP-Glc regeneration, RebM yield in S31D-S195Q was higher than that in *At*SuSy1-S195Q, suggesting the better UDP affinity of *Mc*SuSy_S31D than that of *At*SuSy1 (Table 1). When additional 1 mM UDP was supplemented, *At*SuSy1 with higher activity (Supplementary Table 5) showed its advantages; the RebM concentration in S31D-S195Q was only increased by 1 g/L, while that in *At*SuSy1-S195Q was increased by 4.1 g/L, exceeding the production of RebM by S31D-S195Q. However, the bottleneck limiting the product yield in such reactions was not the enzyme activities but the poor solubility of RebD. As the intermediate product, it formed quickly at high RebE concentrations, and mainly exist as the insoluble precipitate when the reaction speed of further conversion to RebM was limited by the low RebD concentration in the solution. At 10% DMSO, the production of RebM was enhanced (Figure 6), probably due to the improved solubility of RebD. Based on the addition of 10% DMSO, we kept the initial RebE concentration (20 g/L) as used in Figure 6 and increased the number of feeding (three times) as well as the reaction time (total 57 h). Finally, 51.06 g/L RebM was generated from 50 g/L RebE. The results also indicated that both enzymes have good stability under the above conditions. As far as we know, this is the highest concentration of RebM obtained by biotransformation ever reported. The fed-batch synthesis of RebM can be further optimized to achieve a higher yield. RebE, instead of RebA, used as raw material for RebM production, may have an impact on *Stevia* breeding and cultivation.

Thus, *Mc*SuSy is the first characterized SuSy derived from eukaryotic algae, with UDP preference and DMSO tolerance and provides a desirable SuSy candidate to construct the cost-effective SuSy-GT cascade reactions for glycosylation of compounds with low solubility, especially some hydrophobic receptors.

## Data availability statement

The datasets presented in this study can be found in online repositories. The names of the repository/repositories and accession number(s) can be found in this article/Supplementary material.

## Author contributions

YL and HJ conceived and designed the experiment. KC, LL, and RM performed all the research. YL, RM, and LL wrote the manuscript with input from all authors who reviewed the final manuscript. HP, JD, and YT extracted and analyzed the data. YL and LL revised the manuscript. All authors contributed to the article and approved the submitted version.

## Footnotes

1
https://www.ncbi.nlm.nih.gov/Blast.cgi

2
https://www.genome.jp/tools-bin/clustalw

3
https://protein-sol.manchester.ac.uk/

4
http://weblogo.threeplusone.com/

5
http://www.cbs.dtu.dk/services/NetPhos/

6
http://zinc.docking.org/

## References

[B1] Bailey T. L. Elkan C. (1994). Fitting a mixture model by expectation maximization to discover motifs in biopolymers. *Proc. Int. Conf. Intell. Syst. Mol. Biol.* 2 28–36. 7584402

[B2] Baroja-Fernández E. Muñoz F. J. Li J. Bahaji A. Almagro G. Montero M. (2012). sucrose synthase activity in the sus1/sus2/sus3/sus4 *Arabidopsis* mutant is sufficient to support normal cellulose and starch production. *Proc. Natl. Acad. Sci. U.S.A.* 109:321. 10.1073/pnas.1117099109 22184213 PMC3252950

[B3] Blom N. Gammeltoft S. Brunak S. (1999). Sequence and structure-based prediction of eukaryotic protein phosphorylation sites. *J. Mol. Biol.* 294 1351–1362. 10.1006/jmbi.1999.3310 10600390

[B4] Bungaruang L. Gutmann A. Nidetzky B. (2013). Leloir glycosyltransferases and natural product glycosylation: Biocatalytic synthesis of the C-glucoside nothofagin, a major antioxidant of Redbush Herbal Tea. *Adv. Synth. Catal.* 355 2757–2763. 10.1002/adsc.201300251 24415961 PMC3883091

[B5] Chen L. L. Cai R. X. Weng J. Y. Li Y. Jia H. H. Chen K. Q. (2020). Production of rebaudioside D from stevioside using a UGTSL2 Asn358Phe mutant in a multi-enzyme system. *Microb. Biotechnol.* 13 974–983. 10.1111/1751-7915.13539 32011106 PMC7264896

[B6] Chen L. L. Pan H. Y. Cai R. X. Li Y. Jia H. H. Chen K. Q. (2021). Bioconversion of stevioside to rebaudioside E using glycosyltransferase UGTSL2. *Appl. Biochem. Biotechnol.* 193 637–649. 10.1007/s12010-020-03439-y 33057971

[B7] Chen L. L. Sun P. Zhou F. Li Y. Chen K. Q. Jia H. H. (2018). Synthesis of rebaudioside D, using glycosyltransferase UGTSL2 and in situ UDP-glucose regeneration. *Food Chem.* 259 286–291. 10.1016/j.foodchem.2018.03.126 29680056

[B8] Chen T. Y. Chen Z. Y. Wang N. Chu J. L. Fan B. Cheng C. (2021). Highly regioselective and efficient biosynthesis of polydatin by an engineered UGT_BL_1 – *At*SuSy cascade reaction. *J. Agric. Food Chem.* 69 8695–8702. 10.1021/acs.jafc.1c02518 34319737

[B9] Chu J. L. Yue J. H. Qin S. Li Y. Q. Wu B. He B. F. (2021). Biocatalysis for rare ginsenoside Rh2 production in high level with co-immobilized UDP-glycosyltransferase Bs-Yjic mutant and sucrose synthase Atsusy. *Catalysts* 11:132. 10.3390/catal11010132

[B10] Crooks G. E. Hon G. Chandonia J. M. Brenner S. E. (2004). WebLogo: A sequence logo generator. *Genome Res.* 14 1188–1190. 10.1101/gr.849004 15173120 PMC419797

[B11] Curatti L. Porchia A. C. Herrera-Estrella L. Salerno G. L. (2000). A prokaryotic sucrose synthase gene (susA) isolated from a filamentous nitrogen-fixing cyanobacterium encodes a protein similar to those of plants. *Planta* 211 729–735. 10.1007/s004250000343 11089687

[B12] De Bruyn F. Maertens J. Beauprez J. Soetaert W. De Mey M. (2015). Biotechnological advances in UDP-sugar based glycosylation of small molecules. *Biotechnol. Adv.* 33 288–302. 10.1016/j.biotechadv.2015.02.005 25698505

[B13] Desmet T. Soetaert W. Bojarová P. Křen V. Dijkhuizen L. Eastwick-Field V. (2012). Enzymatic glycosylation of small molecules: Challenging substrates require tailored catalysts. *Chem. Eur. J.* 18 10786–10801. 10.1002/chem.201103069 22887462

[B14] Diricks M. De Bruyn F. Van Daele P. Walmagh M. Desmet T. (2015). Identification of sucrose synthase in nonphotosynthetic bacteria and characterization of the recombinant enzymes. *Appl. Microbiol. Biotechnol.* 99 8465–8474. 10.1007/s00253-015-6548-7 25846332

[B15] Diricks M. Gutmann A. Debacker S. Dewitte G. Nidetzky B. Desmet T. (2016). Sequence determinants of nucleotide binding in sucrose synthase: Improving the affinity of a bacterial sucrose synthase for UDP by introducing plant residues. *Protein Eng. Des. Sel.* 30 141–148. 10.1093/protein/gzw048 27590052

[B16] Elling L. (1995). Effect of metal ions on sucrose synthase from rice grains-a study on enzyme inhibition and enzyme topography. *Glycobiology* 5 201–206. 10.1093/glycob/5.2.201 7780195

[B17] Figueroa C. M. Asencion Diez M. D. Kuhn M. L. McEwen S. Salerno G. L. Iglesias A. A. (2013). The unique nucleotide specificity of the sucrose synthase from *Thermosynechococcus elongatus*. *FEBS Lett.* 587 165–169. 10.1016/j.febslet.2012.11.011 23196182

[B18] Guo B. D. Deng Z. W. Meng F. Wang Q. F. Zhang Y. Yuan Z. B. (2022). Enhancement of rebaudioside M production by structure-guided engineering of glycosyltransferase UGT76G1. *J. Agric. Food Chem.* 70 5088–5094. 10.1021/acs.jafc.2c01209 35417157

[B19] Gutmann A. Nidetzky B. (2016). Unlocking the potential of leloir glycosyltransferases for applied biocatalysis: Efficient synthesis of uridine 5’-diphosphate-glucose by sucrose synthase. *Adv. Synth. Catal.* 358 3600–3609. 10.1002/adsc.201600754

[B20] Gutmann A. Lepak A. Diricks M. Desmet T. Nidetzky B. (2017). Glycosyltransferase cascades for natural product glycosylation: Use of plant instead of bacterial sucrose synthases improves the UDP-glucose recycling from sucrose and UDP. *Biotechnol. J.* 12:1600557. 10.1002/biot.201600557 28429856

[B21] Hardin S. C. Winter H. Huber S. C. (2004). Phosphorylation of the amino terminus of maize sucrose synthase in relation to membrane association and enzyme activity. *Plant Physiol.* 134 1427–1438. 10.1104/pp.103.036780 15084730 PMC419819

[B22] Hebditch M. Carballo-Amador M. A. Charonis S. Curtis R. Warwicker J. (2017). Protein-sol: A web tool for predicting protein solubility from sequence. *Bioinformatics* 33 3098–3100. 10.1093/bioinformatics/btx345 28575391 PMC5870856

[B23] Huber S. C. Huber J. L. Liao P. C. Gage D. A. McMichael R. W. Chourey P. S. (1996). Phosphorylation of serine-15 of maize leaf sucrose synthase. Occurrence in vivo and possible regulatory significance. *Plant Physiol.* 112 793–802. 10.1104/pp.112.2.793 8883390 PMC158004

[B24] Katoh K. Misawa K. Kuma K. Miyata T. (2002). MAFFT: A novel method for rapid multiple sequence alignment based on fast Fourier transform. *Nucleic Acids Res.* 30 3059–3066. 10.1093/nar/gkf436 12136088 PMC135756

[B25] Komina O. Zhou Y. Sarath G. Chollet R. (2002). *In vivo* and *in vitro* phosphorylation of membrane and soluble forms of *soybean nodule* sucrose synthase. *Plant Physiol.* 129 1664–1673. 10.1104/pp.002360 12177479 PMC166754

[B26] Krieger E. Koraimann G. Vriend G. (2002). Increasing the precision of comparative models with YASARA NOVA–a self-parameterizing force field. *Proteins Struct. Funct. Genet.* 47 393–402. 10.1002/prot.10104 11948792

[B27] Kulmer S. T. Gutmann A. Lemmerer M. Nidetzky B. (2017). Biocatalytic cascade of polyphosphate kinase and sucrose synthase for synthesis of nucleotide-activated derivatives of glucose. *Adv. Synth. Catal.* 359 292–301. 10.1002/adsc.201601078

[B28] Kumar S. Stecher G. Tamura K. (2016). MEGA7: Molecular evolutionary genetics analysis version 7.0 for bigger datasets. *Mol. Biol. Evol.* 33 1870–1874. 10.1093/molbev/msw054 27004904 PMC8210823

[B29] Mao Z. C. Shin H. D. Chen R. R. Z. (2006). Engineering the *E. coli* UDP-glucose synthesis pathway for oligosaccharide synthesis. *Biotechnol. Progr*. 22 369–374. 10.1021/bp0503181 16599548

[B30] Miller G. L. (1959). Use of dinitrosalicilic acid reagent for determination of reducing sugars. *Anal. Chem.* 31, 426–428. 10.1021/ac60147a030

[B31] Morris G. M. Huey R. Lindstrom W. Sanner M. F. Belew R. K. Goodsell D. S. (2009). AutoDock4 and AutoDockTools4: Automated docking with selective receptor flexibility. *J. Comput. Chem.* 30 2785–2791. 10.1002/jcc.21256 19399780 PMC2760638

[B32] Nidetzky B. Gutmann A. Zhong C. (2018). Leloir glycosyltransferases as biocatalysts for chemical production. *ACS Catal.* 8 6283–6300. 10.1021/acscatal.8b00710

[B33] Pei J. J. Chen A. N. Zhao L. G. Cao F. L. Xiao W. (2019). Synergistic catalysis of glycosyltransferase and sucrose synthase to produce isoquercitrin through glycosylation of quercetin. *Chem. Nat. Compd.* 55 453–457. 10.1007/s10600-019-02712-9

[B34] Ping Q. Yang L. F. Jiang J. J. Yuan J. C. Ai S. Sun S. Q. (2022). Efficient synthesis of rebaudioside D2 through UGT94D1-catalyzed regio-selective glycosylation. *Carbohydr. Res.* 522:108687. 10.1016/j.carres.2022.108687 36270051

[B35] Römer U. Schrader H. Günther N. Nettelstroth N. Frommer W. B. Elling L. (2004). Expression, purification and characterization of recombinant sucrose synthase 1 from *Solanum tuberosum* L. for carbohydrate engineering. *J. Biotechnol.* 107 135–149. 10.1016/j.jbiotec.2003.10.017 14711497

[B36] Saitou N. Nei M. (1987). The neighbor-joining method: A new method for reconstructing phylogenetic trees. *Mol. Biol. Evol.* 4 406–425. 10.1093/oxfordjournals.molbev.a040454 3447015

[B37] Sauerzapfe B. Engels L. Elling L. (2008). Broadening the biocatalytic properties of recombinant sucrose synthase 1 from potato (*Solanum tuberosum* L.) by expression in *Escherichia coli* and *Saccharomyces cerevisiae*. *Enzyme Microb. Technol.* 43 289–296. 10.1016/j.enzmictec.2008.04.001

[B38] Schmölzer K. Gutmann A. Diricks M. Desmet T. Nidetzky B. (2016). Sucrose synthase: A unique glycosyltransferase for biocatalytic glycosylation process development. *Biotechnol. Adv.* 34 88–111. 10.1016/j.biotechadv.2015.11.003 26657050

[B39] Schmölzer K. Lemmerer M. Gutmann A. Nidetzky B. (2017). Integrated process design for biocatalytic synthesis by a Leloir glycosyltransferase: UDP-glucose production with sucrose synthase. *Biotechnol. Bioeng.* 114 924–928. 10.1002/bit.26204 27775150

[B40] Schwab W. Fischer T. Wüst M. (2015). Terpene glucoside production: Improved biocatalytic processes using glycosyltransferases. *Eng. Life Sci.* 15 376–386. 10.1002/elsc.201400156

[B41] Stein O. Granot D. (2019). An overview of sucrose synthases in plants. *Front. Plant Sci.* 10:95. 10.3389/fpls.2019.00095 30800137 PMC6375876

[B42] Sun L. C. Xin F. J. Alper H. S. (2021). Bio-synthesis of food additives and colorants-a growing trend in future food. *Biotechnol. Adv.* 47:107694. 10.1016/j.biotechadv.2020.107694 33388370

[B43] Takeda H. Niikura M. Narumi A. Aoki H. Sasaki T. Shimada H. (2017). Phosphorylation of rice sucrose synthase isoforms promotes the activity of sucrose degradation. *Plant Biotechnol.* 34 107–113. 10.5511/plantbiotechnology.17.0326a 31275015 PMC6543756

[B44] Tao Y. H. Xu J. J. Shao J. L. He X. Y. Cai R. X. Chen K. (2023). Three glycosyltransferase mutants in a one-pot multi-enzyme system with enhanced efficiency for biosynthesis of quercetin-3,4’-*O*-diglucoside. *J. Agric. Food Chem*. 71 6662–6672. 10.1021/acs.jafc.3c01043 37079496

[B45] Terasaka K. Mizutani Y. Nagatsu A. Mizukami H. (2012). In situ UDP-glucose regeneration unravels diverse functions of plant secondary product glycosyltransferases. *FEBS Lett.* 586 4344–4350. 10.1016/j.febslet.2012.10.045 23159939

[B46] Trott O. Olson A. J. (2010). AutoDock Vina: Improving the speed and accuracy of docking with a new scoring function, efficient optimization, and multithreading. *J. Comput. Chem.* 31 455–461. 10.1002/jcc.21334 19499576 PMC3041641

[B47] Wang X. Q. Li J. Q. Li L. N. Zhu L. P. Huang F. (2023). Fusion glycosyltransferase design for enhanced conversion of rebaudioside A into rebaudioside M in cascade. *Mol. Catal.* 547:113317. 10.1016/j.mcat.2023.113317

[B48] Wang Y. Chen L. L. Li Y. Li Y. Y. Yan M. Chen K. Q. (2016). Efficient enzymatic production of rebaudioside A from stevioside. *Biosci. Biotech. Bioch.* 80 67–73. 10.1080/09168451.2015.1072457 26264414

[B49] Wang Z. Y. Liu W. B. Liu W. Ma Y. Y. Li Y. T. Wang B. Q. (2021). Co-immobilized recombinant glycosyltransferases efficiently convert rebaudioside A to M in cascade. *RSC Adv.* 11 15785–15794. 10.1039/d0ra10574k 35481200 PMC9029319

[B50] Wu R. Asencion Diez M. D. Figueroa C. M. Machtey M. Iglesias A. A. Ballicora M. A. (2015). The crystal structure of *Nitrosomonas europaea* sucrose synthase reveals critical conformational changes and insights into the sucrose metabolism in prokaryotes. *J. Bacteriol.* 197 2734–2746. 10.1128/JB.00110-15 26013491 PMC4524045

[B51] Xiao J. B. Muzashvili T. S. Georgiev M. I (2014). Advances in the biotechnological glycosylation of valuable flavonoids. *Biotechnol. Adv.* 32 1145–1156. 10.1016/j.biotechadv.2014.04.006 24780153

[B52] Yu J. Tao Y. H. Pan H. Y. Lin L. Sun J. Y. Ma R. Q. (2022). Mutation of stevia glycosyltransferase UGT76G1 for efficient biotransformation of rebaudioside E into rebaudioside M. *J. Funct. Foods* 92:105033. 10.1016/j.jff.2022

[B53] Zhang L. Gao Y. N. Liu X. F. Guo F. Ma C. X. Liang J. H. (2019). Mining of sucrose synthases from *Glycyrrhiza uralensis* and their application in the construction of an efficient UDP-recycling system. *J. Agric. Food Chem.* 67 11694–11702. 10.1021/acs.jafc.9b05178 31558015

[B54] Zhang T. T. Myint K. Z. Xia Y. M. Wua J. (2021). A comparative study on physicochemical and micellar solubilization performance between monoglucosyl rebaudioside A and rebaudioside A. *J. Sci. Food Agric.* 102 2651–2659. 10.1002/jfa.11604 34687452

[B55] Zhao H. Caflisch A. (2013). Discovery of ZAP70 inhibitors by high-throughput docking into a conformation of its kinase domain generated by molecular dynamics. *Bioorg. Med. Chem. Lett.* 23 5721–5726. 10.1016/j.bmcl.2013.08.009 23993776

[B56] Zhao L. T. Ma Z. B. Wang Q. Hu M. F. Zhang J. X. Chen L. (2023). Engineering the thermostability of sucrose synthase by reshaping the subunit interaction contributes to efficient UDP-glucose production. *J. Agric. Food Chem.* 71 3832–3841. 10.1021/acs.jafc.2c08642 36795895

[B57] Zheng Y. Anderson S. Zhang Y. Garavito R. M. (2011). The structure of sucrose synthase-1 from *Arabidopsis thaliana* and its functional implications. *J. Biol. Chem.* 286 36108–36118. 10.1074/jbc.M111.275974 21865170 PMC3195635

