# Supplementary material for: Identification of sucrose synthase from Micractinium conductrix to favor biocatalytic glycosylation
Source: Front Microbiol. 2023 Aug 15;14:1220208. doi: 10.3389/fmicb.2023.1220208 (PMC10465243; doi:10.3389/fmicb.2023.1220208)
Supplement: Supplementary file 1 [file Data_Sheet_1.pdf]

Supplementary Material

1 Supplementary Data

> DNA coding sequence of *Mc*SuSy (Codon-optimized, with a 6-histidine tag fused at the *C*-terminus)

ATGAGCGCAGGAGCTGATTCTCCCTCAAGTCAACCCTTCTTGGCTAGCCCGCGTGGTGTTATCACCCCGCGTACCTTCACCCGCTCGCTGTCGTTCG
CAGGCGGTACGCCGTCCGAAATTTTGAAGGCGGGCTTGGTTCACAGCCGTAATGAGCTGGTGCTGCTGTTCAGCCGTTGCATGGCAAAGAGCAAAGC
GGACAAGCCAATTCTGTTACCGCATATTATTATGGATGAGTTATGCGCCGTGTGTGATGAATGCAATAATCCGATGCTGAAATCTGGTGAGATCGCG
GCGATCTTGAAAACCGTTCAGGAAGCGGTAGTTATCGCACCGCGAATCGCCTTCGCGTTGAGACCTACGATGGGTGAATGGTACTACGTTCGCGTGT
CTGTTGAAGATATGCGCGTTGAAGAAATGACCGCTGCGCACTATCTCGCGTTTAAAGAGAAGCTGGTTCCACTGGACCAAGATCGTCACGGCTACGA
CCCATTCGTGCTGGAGCTGGACTTGAAGCCGTTTGGCGCACACCAGCCTAAGATTAGCCTGCAAAGCCACATCGGTAACGGTGTTAGCTTCCTGAAT
CGCACGCTGTCGGCGAAAATGTTTTCCCAGAACGCCAATGCGGAAGGCAGCCAATTGATGTTGGACTTCCTGCGTGAATTTAAGCACGGAGGCGAAA
AGTTACTGCTAAGCCCGCGTGTGAATAGCGTGCAGAAATTGCGCCACTCACTGCTGCGTGCTGACCGCTTACTGGAGAAGCACGAAGACGAGGACCC
GCTGTCTGTTGTGCAAGGTATCGACGAGCTGGGCTTCCTGCCGGGTTGGGGTAACACTGTTGGCCGTGTTAGAGAGTCCTTTCAGCTGCTGTTGGAC
ATCATCCAGGCACCAGACGCTGATACTCTGGAAAAGTTCCTGGCCCGCTTGCCGCTGATGGTTAAAGTTGTCATCTTGTCGCCGCATGGTTATTTCG
GTCAAACCAATGTGTTGGGTATGCCGGACACCGGTGGTCAGGTGGTCTACATTCTGGACCAGGTTCGTGCGATGGAGCGTGAGATGCAGCAGCGTCT
AGATGAAGCGGGCCTTCAGAATGTGAAAGCGGATGTTGTTGTGCTGACCCGTCTGATTCCGGATGCACATGGTACTTCCTGCAACGAGCGCTTGGAG
CCGATTAGTGGCTGCCAAAACGCGCGTATCCTGCGTGTTCCGTTTCGGGATAGCGAAGGTCGTATCCTGAATCATTGGGTTTCCAGGTTCGATCTGT
GGCCGTATCTGGAGCGCTTTACCATTGACGCCACGAAGGAAATCCTGGCTGAAATGGGCGGTAAACCGGACTTCATTATCGGCAACTATAGCGATGG
TAACTTAGTGGCGACCCTCATGAGCCATCGTATGAACGTGACCCAGTGTAACATTGCGCATGCGCTGGAAAAAACCAAATACGATGATGCCGACATT
TACTGGCAGAAGCTTGAAGACAAATACCATTTTAGCTGCCAATTTACGGCCGACCTGATCGCAATGAACTCTGCGGATTTTATCGTCACCTCCACCT
ACCAGGAGATCGCTGGTCATGAGGAAATGGTGGGCCAATATGAGAGCTATAAGAGCTTTACCATGCCACAGCTGTACCGCGTTGTGGAGGGCATTGA
CATCTACAACCCGAAGTTCAACATTGTGAGCCCGGGTGCGGACCTGGACATCTACTTTCCGTACCAGGAGAAGGAGAGGCGTTTGACCGGCCTGCAT
AAGGACATTGAAGCGCTGCTGTTTGACCCGGATTTTAAAGGTACGGTCGGTCAGTTAGAGGACAGAGACAAACCGATCCTGTTTTCTATGGCGCGTT
TGGACAAAGTGAAGAACCTGACTGGCCTGGCCGAGTGGTACGCGGGCAATCAGCGTTTGCGTGGTCTGGTCAACCTGGTCATAGTCGGTGGCGTTAT
TGATCCGGCTGCTACGATGGACCGCGAAGAGGCAGCGGAGTGCGAACACATGCACGAACTTGTCGAGAAGTATAAAATGCATGGCACGTTCCGCTGG
ATTGTTGCTCAGAAGAACCGTGTACGTAACGGCGAGCTGTATAGATACATCGCTGATACTCGTGGTGCTTTCGCCCAACCGGCACTGTACGAAGCCT
TTGGGCTGACCGTGATCGAGGCGATGACCTGTGGTCTTCCGACCTTCGCAACCAACCACGGCGGTCCGTCCGAAATCATTAAACACAAGAAAAGCGG
CTTCCATATCGACCCGTATCACGGTGCGGAAGCGGCGGATCTGATGGCGGATTTCTTTGAACGTTCTCAAAAGGAGCCGTCTCATTGGACCAAGATC
TCCGAGGCGGCGCAAGAACGTATTTTTAGCCGTTACACGTGGTCAATCTACGCAAAGCGCCTGGTGACCTTGAGCCACGTGTATACCTTCTGGAAAC
ACGTGACCAGCCTGGAGAGCCGCGAAACCAAACGTTATCTGGAGATGTTCTATATTCTGCAAATGCGTAAATTGGTAGCCAAAATGTCGGAAGAGAC
CGTGGAAAAGGAGAAGGCGGCGGCAGAGGCAGGTCCGGCGGGCCCGCCTAAAGTTGGCTTTGGTGCTATGGTTGAGCACCATCACCACCACCACTAA

> DNA coding sequence of *Cb*SuSy1 (Codon-optimized, with a 6-histidine tag fused at the *C*-terminus)

ATGGAGTATGCGGCAGAAGATGCTATGACAATGACCGTGGATCTGGAGGACGGGGAACTCAAGCCGCGTCCGGAAAAAATGCCAGAGCTCTCTCGCA
TCGCGAGCATGACTGTGCGTGTGAAGGACAGCATCCGTGATTATCGCAACCAGATTATCTTTATGTTGTCCAGACTGGTGGAAAAAGGTAAGCACAC
CCTGCAGCCGCATGAACTCCAAAAGCAGCTGGAGCGCGTGGGTGCCATCGAGTGCTTTAGCGGTACGACCATCAAGGACAGCGCCTTCGCGACCCTG
TTGCAAAGCGCGCAAGAAGCAGTGGTGATCCCGCCTTGGATTGCAATGGCCGCGCGTCCGCGTGTGGCCGAATGGCTGTATGTTCGTATCAACGTTT
TCGAACTGTCCGTGGACGAGCTGACCGTTTCAGAATACTTGGATTTTAAAGAACAGCTGAAGCTGGACAAACCGGTTGACGAGTTCAGCCTGTTGGA
GTTTGACATGGGTCCGTTTAACGCTAATTTTCCGCGTATGACTCGTCCGAGCTCGATCGGCAATGGTGTTGAGTTCCTGAATAAACACCTGTCCACC
AAGCTGTTCAAGAACGCGAAGGCGCTCCAACCGCTGTTAGACTTCCTGCGTAATCACAAATACCAGGGTGAGACGCTGATGGTTAACGATCAGCTGG
AGGACCTGCCAGCGCTGCGTGATGGTTTGAAAAAGGCCACCGAATACCTCTCCTCTGTCGGTCATGATGCGCCGGTTTCTGCGGTGCAAGATCAGTT
ACGAGCACTGGGCTTCGAGAACGGCTGGGGTAATTGTGCAGGCCGTATTAAAGATATGATGGAATTGCTGGAGGACCTGATGCAGGCACCGAGCCCA
GTACTGTTGGAGAAGTTCCTGGCACGCGTGCCAATGATTTTCAACGTTGCGATCATCAGCCCGCACGGCTATTTTGGCCAAGCGAACGTGCTGGGTC
TGCCGGACACCGGTGGTCAGGTTGTGTATATCCTAGATCAAGTTCGTGCTTTGGAGCGCGAAATGCTGAACCACGTTCAGAACCAAGGTTTGCGTTT
TAAACCGCAGGTCATCGTGTTGACGCGTTTGGTTCCGGACGCACACGGCACGAACTGCGATCAACGTCTGGAGAAGATCGAGGGCACTGAATACGCG

AAAATTCTGCGTGTTCCGTTCCGCGACCTGGCTAAGGGCGAAGGTATTCTGCGCAAATGGGTTTCGCGTTTTGATATCTGGCCGTACCTGGAAACGT
TCGCCGAAGATTCAGCGAAGGCGTTAGTAGAGGAGATGGGCGGCAATCCGGACCTGATCATCGGCAACTATAGCGACGGCAATTTGGTCGCGACCCT
ACTCTCCCATCGTATGCAGGTTACCCAGTGTACCATTGCGCATGCTCTGGAAAAGACCAAGTACCCGAGCAGCGATGTTAACTGGAAAGAAGTGGAA
GAAAAGTACCATTTTAGCTGTCAGTTTACCGCAGATCTGATTGCCATGAATCACACCGATTTCATCGTTACCAGCACCTATCAAGAAATAGCGGGCG
GAATTGACACGGTCGGTCAATATGAAAGCCATCAGGCGTTCACCATGCCGGGTCTCTATCGTGTTGTGAACGGCATTAACGTGTTCGACCCGAAATT
CAATATTGTGGCTCCGGGCGCAGATGCGGAGGTTTACTTCCCGTATACCGCGAAAGAGCGCCGTTTAACCACCTTCCATAGCGCGATCGAAGATTTG
TTGTTCGGTAACATGGAAGAGCCGGCACTTTGCAAAAGCGTGATTAAAAACCGTCATAAGCCTATCCTTTTTCTATGGCTCGCCTGGACAAGGTTA
AGAACCTGACCGGACTGGTTGAGATGTTCGGCAAGAACCAGCGATTGAGACGCCTGGTCAACTTAGTAGTTATTGGCGGCTACATCGATCCTACTCT
GTCGAAAGACCGTGAGGAAGTGGAGCAGATTAATCTGATGCATAAACTGATTGAGAAGTACCAACTGAATGGCGACATGCGCTGGATTGTTGCTCAG
AAAAATCGTGTGCGTAACGGTGAGTTATACCGCTACGTAGCGGACACCCGTGGTGCGTTTGTTCAGCCGGCATTCTATGAGGCGTTCGGTCTGACCG
TCGTGGAGGCCATGACGTGCGGTTTGCCGACCTTTGCTACGTGCCACGGTGGCCCGGCGGAAATCATTGAGGACGGCAAGAGCGGCTTCCACATTGA
TCCGTACCAGCCGGACGAGACCGCCAAGGCCCTAGGTGACTTTTTTGAAGCGGCTGCTGCGGACCCGACGAAGTGGGAAGCGGTCAGCCGCGGTGGT
TTGGAACGCATTCGCAGCAAATACACCTGGGAGATCTATGCTCGTCGTCTTATGACCCTGAGTCGTGAATATGGTTTTTGGAAATTCGTGTCCGATC
TGGATCGCAGAGAAGCTAAACGTTATCTGGAAATGTTTTACATCTTGAAATACCGCCCACTGGTGAAAAAAGTTCCGCTGACAGTCGACGCACCGGA
CAGGCCGATGGCAGGTCGTAGAGTTGAGCACCACCACCACCACCACTAA

> DNA coding sequence of *Cb*SuSy2 (Codon-optimized, with a 6-histidine tag fused at the *C*-terminus)

ATGGGAGCTGATCTAGTACCCAGGCCAGAAAAGCTACCGGAACTGACCCGTATGCATAGCATGACCGATCGTGTTAAAGGCAGCATTGCAGAGTATC
GTAATCAGGTGATCCTGCTGCTCTCCCGTTATGTTTCCAACGGCAAACACACCCTGCAGCCTCATGAACTGAAAAATGAATTGGAGCGCGTGGCCGA
GCTGGAGTGCTTTGCCGGCACCCAAATTAAGGATAGCGCGTTCGCTAAGATCCTGCGCGCGACCCAAGAGGCGGTTGTAATCCCACCGTATATTGCG
CTGGCGGTTCGTCCGCGTGTTGCGGAGTGGCAGTACCTGCGCGTCAACGCGTTCGAGATGACGGTGGAAGAATTGTCCCCGAGCGAATACCTGGAGT
TCAAAGAGAGATTGAAGGCGGCAGATGATGAAGCGCCACCGATCTGTAGCGACTTCGCAACCCTTGAGATCGACATGGAGCCGTTTAATGCTAGCTT
TCCGCGTCTGACTCGCCCGTCGTCCATTGGCGATGGCGTCAGCTATCTGAACAAACACCTGTCTAGCCGTATGTTTAAGGAAGCGGGCGGTCTGCAA
CCGCTGCTGGATTTCCTGCGCACCCACAAATGCGTTGGCGAGACGCTGATGCTGAACGCACGCATCGACACCTTGGAGAAGCTGCGCAGCAACCTAG
CTAAAGCGGAGGAATTTTTGGGTGCGCTGCCGGCAGACACGCCAGTGGGCAGCGTGGCGACGCGTCTCCAAGAACTGGGTTTTGAACGCGGCTGGGG
TGATACTGCGGGTCGCATCAAAGACATGGTAGATATGCTGTCCGATTTAATGCAGGCTCCGGACGCCGACCTGTTGGAAAAGTTCTTGGGCCGTATC
CCGGTGATTTTTAACGTGGCGATTATGAGCCCGCACGGTTACTTCGGACAAGCAAATGTTCTGGGCCTGCCTGATACTGGTGGTCAAGTCGTGTATA
TTCTGGATCAGGTTAAAGCCTTGGAGCGTGATCTGCTGCATCACAGCAAGCAGCAGGGTTTGAACTTTAAGCCGCAGATAATCGTTCTGACCCGCCT
GATTCCGGATGCGCACGGCACCAGTTGCAATCAAAGAATCGAGCACATTGACGGCACCCAATACAGCAAGATTCTGCGTGTGCCGTTTAAGAACCCG
AAGGATGGTTCTGTTTTGCGTAAGTGGGTTTCGCGTTTTGACGTGTGGCCATATATGGAGCAGTTCACGGAAGATAGCGTGCACGAATTGCGTGCTG
AATTTGGTGGCAACCCGGACTTGATTATCGGCAACTATAGCGACGGCAACCTGGTTGCTGTCCTGCTGGCGCATCGTCTGAAGGTGACTCACTGCAC
CATTGCACACGCGTTGGAGAAGACGAAGTATCCGAACAGCGACCTGAATTGGAAAGAACTTGACGAGAAGTACCATTTTTCTTGCCAGTTTACTGCC
GACCTGATCGCTATGAATCATGCGGATTTCATTATCACCTCCACCTATCAAGAAATCGCGGGTCGTGCGGATGCAGTTGGTCAATACGAGAGCCATC
AGGCATATACCATGCCGGGTTTGTACAGAGTCGTGAACGGTATTGACGTCTTTGACCCAAAATTCAATATTGTGTCCCCGGGTGCTGACGCTGACAC
CTACTACCCGTACTTCATTAAGGAGAAACGTCTCACGGCGTTCCATCCGGAAATCGAGGAGCTGCTTTACGGCCAGAAAGAAGATGTTCGTCTTTGC
CGTGGTGTTCTGCAAGATCGTAGCAAGCCGATCATCTTCACGATGGCAAGATTAGACAAAGTTAAAAACCTGACCGGTCTTGCCGAGATGTATGGTA
AAAGCGCGCGTCTCCGCAAATTGGTGAACCTGGTGATCGTTGGCGGTTACATCGACCCGAGTCTGAGCATGGACCGCGAGGAAGTTCATCAGATTAA
CCAACTGCATGCCATTATTGACAAATACGCGCTTGACAAGGGAGACATGCGTTGGATTGTGGCGCAGAAACATCGTATGCGTAATGGTGAGATGTAT
CGTTACATCGCCGACACCCGTGGTGCGTTCATCCAGCCGGCTTTTTACGAAGCGTTCGGCCTGACCGTAGTTGAAGCTATGACCTCTGGTTTACCGA
CCTTCGCTACCTGTCATGGCGGGCCTGCGGAAGTGATCAAGCACGGCGTGTCTGGTTACCATATTGATATGTATCGCCCGGATGAAGTTGCCGACTT
GATTGCAGACTTCTTTGAACGCTGCAAAACCGATCCGGGTGAGTGGGACGGTCTGTCCAAGGCTGGCTTGGAACGTATCTATAGCAAATTCACCTGG
GAGATTTATGCGGAGCGCCTGATGACCCTGAGCCGAGTGTACACGTTTTGGAAATTCGTGTCTAACCTCGAACGCAGGGAAGCGCGTCGTTACATCG
AGATGTTCTATAACCTGAAATACCGCCAGTGTGTTAAGACCGTACCGCTTGCGGTGGAGCACCACCACCACCACCACTAA

2 Supplementary Figures and Tables

2.1 Supplementary Figures

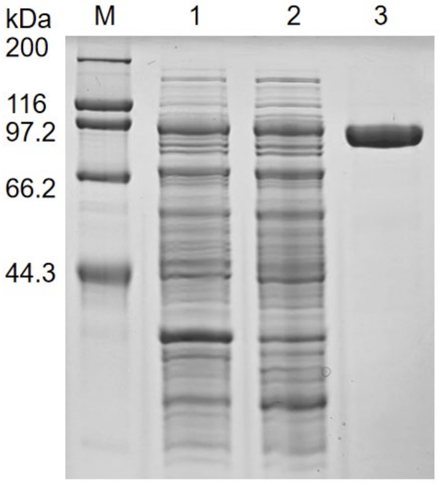

Supplementary Figure 1. SDS-PAGE analysis of the *Mc*SuSy. M: protein markers; Lanes 1: the soluble fraction of cell lysate; Lane 2: the insoluble fractions of cell lysate; Lane 3: the purified *Mc*SuSy.

(A)

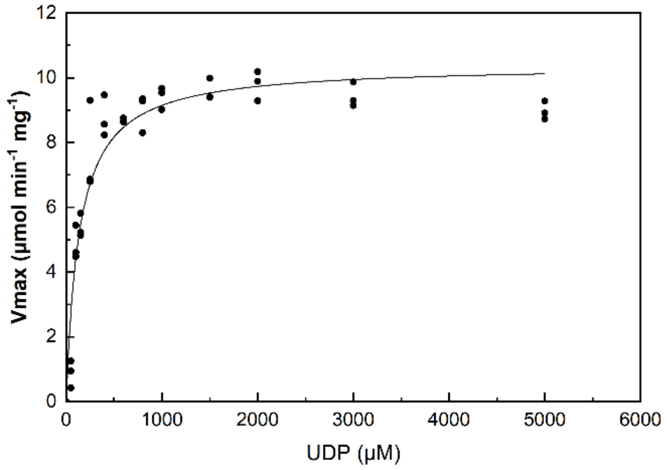

(B)

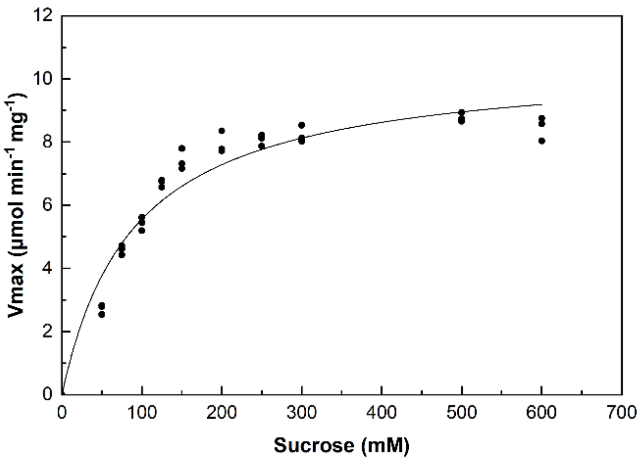

Supplementary Figure 2. The influence of the UDP (A) and sucrose (B) concentrations on the initial rates of *Mc*SuSy.

(A)

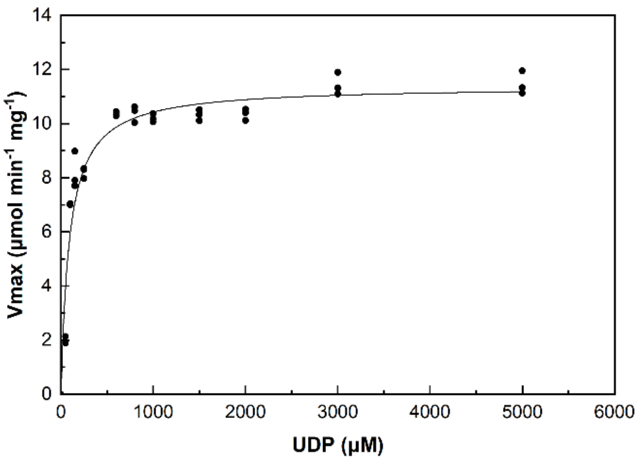

(B)

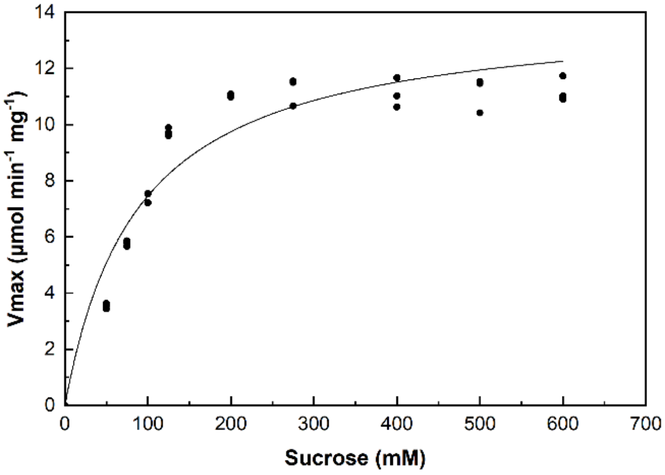

Supplementary Figure 3. The influence of the UDP (A) and sucrose (B) concentrations on the initial rates of *Mc*SuSy_S31D.

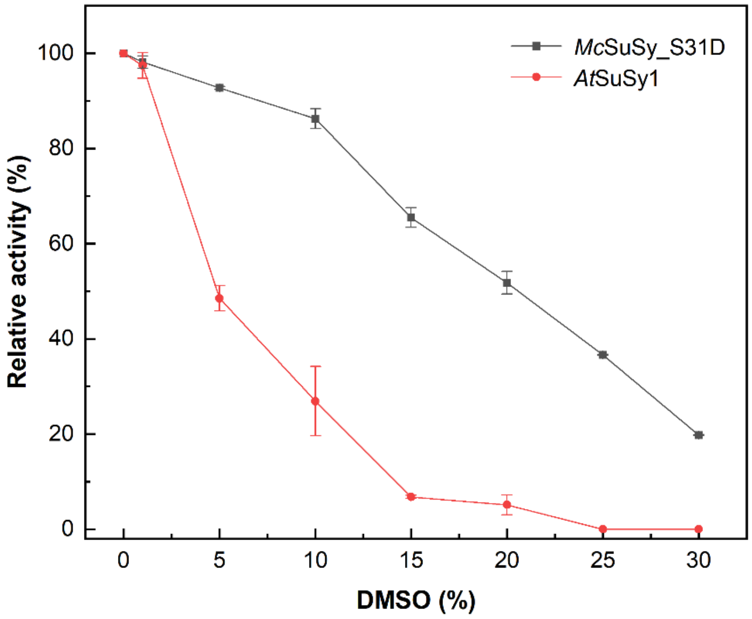

Supplementary Figure 4. The influence of DMSO concentrations on the activities of *Mc*SuSy_S31D and *At*SuSy1. The SuSy assay solutions (100 uL) consisted of 200 mM sucrose, 5 mM UDP, appropriate crude extract, various concentrations of DMSO (v/v) and 50 mM HEPES buffer (pH 7.0). The relative activity (%) was calculated in terms of that of the maximum activity (100%).

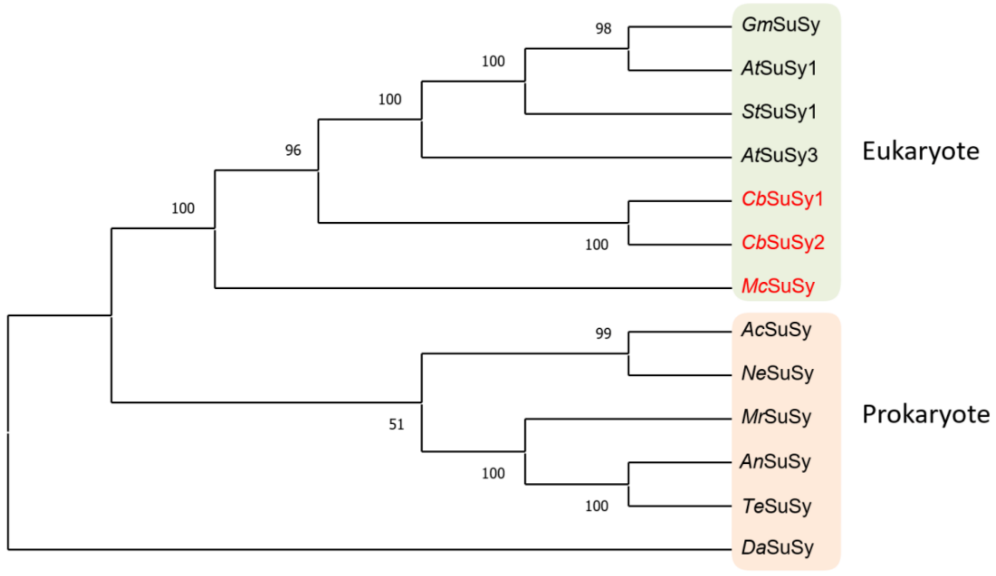

Supplementary Figure 5. Phylogenetic tree constructed using SuSy sequences from different species.

2.2 Supplementary Tables

Supplementary Table 1. Primers used for the site-directed mutagenesis of *Mc*SuSy

Mutant	Primer Sequence
S7D	5'-GGAGCTGATGATCCCTCAAGTCAACCCTTCTTGGCTAGC-3'
	5'-ACTTGAGGGATCATCAGCTCCTGCGCTCATGGTATATCT-3'
S7E	5'-GGAGCTGATGAACCCTCAAGTCAACCCTTCTTGGCTAGC-3'
	5'-ACTTGAGGGTTCATCAGCTCCTGCGCTCATGGTATATCT-3'
T22D	5'-GGTGTTATCGATCCGCGTACCTTCACCCGCTCGCTG-3'
	5'-AGGTACGCGGATCGATAACACCACGCGGGCTAGCCA-3'
T22E	5'-GGTGTTATCGAACCGCGTACCTTCACCCGCTCGCTG-3'
	5'-AGGTACGCGGTTCGATAACACCACGCGGGCTAGCCA-3'
S31D	5'-TCGCTGGATTTCGCAGGCGGTACGCCGTCCGAAATT-3'
	5'-TGCGAAATCCAGCGAGCGGGTGAAGGTACGCGGGGTGATA-3'
S31E	5'-TCGCTGGAATTCGCAGGCGGTACGCCGTCCGAAATT-3'
	5'-TGCGAATTCCAGCGAGCGGGTGAAGGTACGCGGGGTGATA-3'

Note: The codons expected to mutate are underlined.

Supplementary Table 2. Summary of purification of *Mc*SuSy from the recombinant *E. coli* BL21(DE3)

Step	Total protein (mg)	Total activity (U)	Specific activity (U/mg)	Yield (%)
Crude extract	96	68.16	0.71	100
Ni-NTA column purification	0.48	4.51	9.39	6.62

Supplementary Table 3. Prediction of phosphorylation sites at the *N*-terminus of the selected SuSys

SuSy	Site	Residue	Score (>0.9)
*Mc*SuSy	7	S	0.995
*Mc*SuSy	22	T	0.974
*Mc*SuSy	31	S	0.927
*Gm*SuSy	11[a]	S	0.994
*Zm*SuSy	11	S	0.903
*Zm*SuSy	15[b]	S	0.996

Note: [a] The site has been identified by Nakai et al.; [b] The site has been identified by Hardin et al.

Supplementary Table 4. Conserved active site residues in retaining GT-B glycosyltransferases

Enzyme	Site 1	Site 2	Site 3	Site 4
*At*SuSy1	dt**GG**^{303}qvvy	tia**H**^{438}alek	ma**R**^{580}ldrvK^{585}nls	aly**E**^{675}afgl
*An*SuSy	dt**GG**^{295} qvvy	nia**H**^{434}alek	ma**R** ^{576}ldri**K**^{581}nlt	aly**E**^{672} afgl
*Mr*SuSy	dt**GG**^{285} qvvy	gia**H**^{421}alek	ms**R**^{563}ldri**K**^{568}nis	aly**E**^{659} afgl
*Mc*SuSy	dt**GG**^{333} qvvy	nia**H**^{473}alek	ma**R**^{614}ldkv**K**^{619}nlt	aly**E**^{710} afgl
*Cb*SuSy1	dt**GG**^{319} qvvy	tia**H**^{456}alek	ma**R**^{599}ldkv**K**^{604}nlt	afy**E**^{696} afgl
*Cb*SuSy2	dt**GG**^{329} qvvy	tia**H**^{467}alek	ma**R**^{610}ldkv**K**^{615}nlt	afy**E**^{706} afgl

Note: The conserved residues in GT-B glycosyltransferases are in bold capital letters. Residue numbers come from the multiple sequence alignment.

Supplementary Table 5. The specific activities of UGT76G1_S195Q and SuSys in the crude extracts prepared from different recombinant strains.

Strains for enzyme preparation	Enzyme activity (mU/mg)	
	GT	SuSy
E. coli BL21(pRSF-*At*SuSy1-S195Q)	251.22±1.93	679.94±3.23
E. coli BL21(pRSF-S31D-S195Q)	200.92±1.17	368.28±0.21

References

Nakai, T., Konishi, T., Zhang, X., Chollet, R., Tonouchi, N., Tsuchida, T., et al. (1998). An increase in apparent affinity for sucrose of mung bean sucrose synthase is caused by in vitro phosphorylation or directed mutagenesis of Ser11. *Plant Cell Physiol.* 39, 1337-1341. doi: 10.1093/oxfordjournals.pcp.a029339

Hardin, S. C., Winter, H., Huber, S. C. (2004). Phosphorylation of the amino terminus of maize sucrose synthase in relation to membrane association and enzyme activity. *Plant Physiol.* 134, 1427-1438. doi: 10.1104/pp.103.036780
